# Prostaglandin E_2_ Induction during Mouse Adenovirus Type 1 Respiratory Infection Regulates Inflammatory Mediator Generation but Does Not Affect Viral Pathogenesis

**DOI:** 10.1371/journal.pone.0077628

**Published:** 2013-10-16

**Authors:** Mary K. McCarthy, Rachael E. Levine, Megan C. Procario, Peter J. McDonnell, Lingqiao Zhu, Peter Mancuso, Leslie J. Crofford, David M. Aronoff, Jason B. Weinberg

**Affiliations:** 1 Department of Pediatrics and Communicable Diseases, University of Michigan, Ann Arbor, Michigan, United States of America; 2 Department of Microbiology and Immunology, University of Michigan, Ann Arbor, Michigan, United States of America; 3 Department of Internal Medicine, University of Michigan, Ann Arbor, Michigan, United States of America; 4 Department of Environmental Health Sciences, University of Michigan, Ann Arbor, Michigan, United States of America; 5 Department of Internal Medicine, Vanderbilt University, Nashville, Tennessee, United States of America; University of California Los Angeles, United States of America

## Abstract

Respiratory viruses cause substantial disease and are a significant healthcare burden. Virus-induced inflammation can be detrimental to the host, causing symptoms during acute infection and leading to damage that contributes to long-term residual lung disease. Prostaglandin E_2_ (PGE_2_) is a lipid mediator that is increased in response to many viral infections, and inhibition of PGE_2_ production during respiratory viral infection often leads to a decreased inflammatory response. We tested the hypothesis that PGE_2_ promotes inflammatory responses to mouse adenovirus type 1 (MAV-1) respiratory infection. Acute MAV-1 infection increased COX-2 expression and PGE_2_ production in wild type mice. Deficiency of the E prostanoid 2 receptor had no apparent effect on MAV-1 pathogenesis. Virus-induced induction of PGE_2_, IFN-γ, CXCL1, and CCL5 was reduced in mice deficient in microsomal PGE synthase-1 (mPGES-1^-/-^ mice). However, there were no differences between mPGES-1^+/+^ and mPGES-1^-/-^ mice in viral replication, recruitment of leukocytes to airways or lung inflammation. Infection of both mPGES‑1^+/+^ and mPGES-1^-/-^ mice led to protection against reinfection. Thus, while PGE_2_ promotes the expression of a variety of cytokines in response to acute MAV-1 infection, PGE_2_ synthesis does not appear to be essential for generating pulmonary immunity.

## Introduction

Eicosanoids are lipid mediators generated by the release of arachidonic acid from cell membrane phospholipids in response to diverse stimuli. Prostaglandins (PGs) are derived from the oxidation of arachidonic acid by cyclooxygenase (COX) enzymes. Modification of arachidonic acid by COX forms the unstable intermediate molecule PGH_2_, which is converted by specific synthases to form various PGs such as thromboxane, PGD_2_, PGE_2_, PGF_2α_, and prostacyclin (PGI_2_). At least three different synthases have been shown to catalyze the conversion of PGH_2_ to PGE_2_ *in vitro*: microsomal prostaglandin E_2_ synthase (mPGES)-1, mPGES-2, and cytosolic PGES (cPGES/p23) [1-3]. However, neither mPGES‑2 nor cPGES is required for *in vivo* PGE_2_ synthesis [4-6] and mPGES-1 is solely responsible for both basal and inducible PGE_2_ levels *in vivo* [7,8]. 

PGE_2_ regulates immune function in many ways that are likely to affect viral pathogenesis (reviewed in 9). For example, PGE_2_ promotes inflammation through vasodilatory mechanisms, leading to edema and facilitating passive leukocyte recruitment. Additionally, PGE_2_ augments production of the proinflammatory cytokine IL-6 by leukocytes [10] and airway epithelial cells [11]. In regard to adaptive immunity, PGE_2_ exerts an immunosuppressive effect at high concentrations by inhibiting production of the Th1 cytokines interferon (IFN)-γ and IL-12 [12,13]. However, nanomolar concentrations of PGE_2_ enhance Th1 cytokine secretion and differentiation *in vivo* [14,15]. PGE_2_ plays an important role in optimal antibody synthesis. COX inhibitors suppress antibody production in activated human B lymphocytes [16,17], and PGE_2_ can act on uncommitted B lymphocytes to promote isotype switching to IgE or IgG1 [18-20]. PGE_2_ production increases *in vitro* and *in vivo* in response to many respiratory viruses, including respiratory syncytial virus (RSV) [21-24], influenza [25-27], human cytomegalovirus [28] and rhinovirus [29]. During RSV or influenza infection, pharmacologic inhibition of COX enzymes or a genetic deficiency of COX‑2 decreases virus induction of pro-inflammatory cytokine production and pulmonary inflammation [22,30]. 

Adenoviruses are non-enveloped double-stranded DNA viruses that are common causes of respiratory infection [31]. HAdV-5 and recombinant HAdV-5-based vectors induce COX-2 expression and PGE_2_ release in murine fibroblasts [32] and in human primary synovial fibroblasts [33] *in vitro*, respectively. However, little else is known about the role of PGE_2_ in the pathogenesis of adenoviruses or other viruses that commonly cause respiratory infection. Since species-specificity of adenoviruses complicates animal studies with a human adenovirus, we previously established mouse adenovirus type 1 (MAV-1, also known as MAdV-1) as a model to study the pathogenesis of adenovirus respiratory infection in the natural host of the virus [34-40]. Antibodies have a crucial role in preventing severe disseminated MAV-1 infection. Mice lacking B cells or Bruton’s tyrosine kinase (Btk) have increased susceptibility to MAV-1, and antiserum from immune Btk^+/+^ mice protects Btk^-/-^ mice [41]. T cells cause acute immunopathology and are required for long-term host survival following intraperitoneal (i.p.) MAV-1 infection. We previously demonstrated that lung viral loads in mice rechallenged with MAV-1 28 days following primary infection remain at or below the limit of detection [35], indicating that adaptive immune responses to MAV-1 are protective.

 Because previous studies of other respiratory viruses used COX-deficient animals or COX inhibition, their results could be attributed to deficiency of PGE_2_ or other COX-derived mediators. We hypothesized that PGE_2_ production is necessary for the appropriate coordination of inflammatory responses after adenovirus respiratory infection. To test this hypothesis, we evaluated the role of PGE_2_ after MAV-1 respiratory infection using mice deficient in the terminal PGE_2_ synthase, mPGES-1. Consistent with our hypothesis, induction of pro-inflammatory cytokines was reduced in mPGES-1-deficient mice following MAV-1 infection compared to mPGES‑1^+/+^ mice. However, PGE_2_ deficiency did not affect virus-induced lung inflammation, viral replication, or the development of protective immunity in this model.

## Materials and Methods

### Ethics Statement

All animal work was conducted according to relevant national and international guidelines. All animal studies were approved by the University of Michigan Committee on Use and Care of Animals (Protocol Number 9054).

### Mice

mPGES-1 heterozygous mice on a DBA1lac/J background [6] were originally obtained from Pfizer, Inc. (Groton, CT) and then backcrossed onto a C57BL/6 background. Homozygous mPGES-1^-/-^ mice and homozygous wild type mPGES-1^+/+^ mice derived from the same heterozygous mPGES-1^+/-^ parents were bred at the University of Michigan. MHC class II deficient mice (Aβ^-/-^) [42] were purchased from Taconic and bred at the University of Michigan. Adult (4 to 6 weeks of age) males were used in all experiments. All mice were maintained under specific pathogen-free conditions.

### Virus and Infections

MAV-1 was grown and passaged in NIH 3T6 fibroblasts, and titers of viral stocks were determined by plaque assay on 3T6 cells as previously described [43]. Adult mice were anesthetized with ketamine and xylazine and infected intranasally (i.n.) with 10^5^ plaque forming units (p.f.u.) of MAV-1 in 40 μl of sterile phosphate-buffered saline (PBS). Control mice were mock infected i.n. with conditioned media at an equivalent dilution in sterile PBS. Mice were euthanized by pentobarbital overdose at the indicated time points. Lungs were harvested, snap frozen in dry ice, and stored at -80°C until processed further. In separate experiments, mice received an i.p. injection of indomethacin (1.2 mg/kg in PBS) or vehicle control (DMSO similarly diluted in PBS) starting on the day of infection and then on each day thereafter. 

### Histology

Lungs were harvested from a subset of mice and fixed in 10% formalin. Prior to fixation, lungs were gently inflated with PBS via the trachea to maintain lung architecture. After fixation, organs were embedded in paraffin, and 5 µm sections were obtained for histopathology. Sections were stained with hematoxylin and eosin to evaluate cellular infiltrates. All sectioning and staining was performed by the Pathology Cores for Animal Research in the University of Michigan Unit for Laboratory Management. Slides were viewed through a Laborlux 12 microscope (Leitz). Digital images were obtained with an EC3 digital imaging system (Leica Microsystems) using Leica Acquisition Suite software (Leica Microsystems). Final images were assembled using Adobe Illustrator (Adobe Systems). Adjustments to the color balance of digital images were applied in Adobe Illustrator equally to all experimental and control images.

To quantify cellular inflammation in the lungs, slides were examined in a blinded fashion to determine a pathology index as previously described [35], generating separate scores for the severity of cellular infiltrates around airway lumens and interstitial infiltrates (Table 1). Each score was multiplied by a number reflecting the extent of involvement in the lung (5% to 25% = 1, >25% to 50% = 2, >50% = 3). The final pathology index was obtained by adding together the values for cellular infiltrates around airway lumens and for interstitial infiltrates.

**Table 1:** Quantification of cellular inflammation in histologic specimens.

**Score^a^**	**Cellular Infiltrates Around Airway Lumens**	**Interstitial Infiltrates**
0	No infiltrates	No infiltrates
1	1 to 3 cell diameters thick	Increased cells visible only at high power
2	4 to 10 cell diameters thick	Easily seen cellular infiltrates
3	>10 cell diameters thick	Extensive consolidation by inflammatory cells

a A score from 0 to 3 was given for each of the two categories. The score for each category was multiplied by a number reflecting the extent of involvement in the specimen (5% to 25% = 1, >25% to 50% = 2, >50% = 3). The final pathology index score was obtained by adding together values for each category, resulting in a total score that could range from 0 to 18.

### Isolation of DNA and RNA

DNA was extracted from the middle lobe of the right lung using the DNeasy® Tissue Kit (Qiagen Inc.). DNA was extracted from approximately one-fifth of the spleen using the DNeasy® Tissue Kit. For DNA extraction from brain, half of each brain was homogenized using a sterile razor blade, and a portion of the homogenate was used to extract DNA using the DNeasy® Tissue Kit. Total RNA was extracted from lungs as previously described [38]. 

### Analysis of Viral Loads

MAV-1 viral loads were measured in organs using quantitative real-time polymerase chain reaction (qPCR) as previously described [35,38]. Primers and probe used to detect a 59-bp region of the MAV-1 E1A gene are detailed in Table 2. Five μl of extracted DNA were added to reactions containing TaqMan II Universal PCR Mix with UNG (Applied Biosystems), forward and reverse primers (each at 200 nM final concentration), and probe (200 nM final concentration) in a 25 µl reaction volume. Analysis on an ABI Prism 7300 machine (Applied Biosystems) consisted of 40 cycles of 15 s at 90°C and 60 s at 60°C. Standard curves generated using known amounts of plasmid containing the MAV-1 EIA gene were used to convert cycle threshold values for experimental samples to copy numbers of EIA DNA. Results were standardized to the nanogram (ng) amount of input DNA. Each sample was assayed in triplicate. The limit of detection of this assay is typically between 10^1^ and 10^2^ copies of MAV-1 genome per 100 ng input DNA. 

**Table 2:** Primers and probes used for real-time PCR analysis.

**Target**	**Oligonucleotide**	**Sequence (5′ to 3′)**
MAV-1 E1A	Forward primer	GCACTCCATGGCAGGATTCT
	Reverse primer	GGTCGAAGCAGACGGTTCTTC
	Probe	TACTGCCACTTCTGC
IFN-γ	Forward primer	AAAGAGATAATCTGGCTCTGC
	Reverse primer	GCTCTGAGACAATGAACGCT
COX-1	Forward primer	CTTCTTAGGGAATCCCATCTG
	Reverse primer	CTTCAGTGAGGCTGTGTTGACAAG
COX-2	Forward primer	TGACCCCCAAGGCTCAAAT
	Reverse primer	GAACCCAGGTCCTCGCTTATG
TNF-α	Forward primer	CCACCACGCTCTTCTGTCTAC
	Reverse primer	AGGGTCTGGGCCATAGAACT
GAPDH	Forward primer	TGCACCACCAACTGCTTAG
	Reverse primer	GGATGCAGGGATGATGTTC

### Analysis of Host Gene Expression

Cytokine gene expression was quantified using reverse transcriptase (RT)-qPCR. First, 2.5 μg of RNA were reverse transcribed using MMLV reverse transcriptase (Invitrogen) in 20 µl reactions according to the manufacturer’s instructions. Water was added to the cDNA product to bring the total volume to 50 µl. cDNA was amplified using duplexed gene expression assays for mouse CCL5, CXCL1 and GAPDH (Applied Biosystems). Five µl of cDNA were added to reactions containing TaqMan Universal PCR Mix and 1.25 µl each of 20X gene expression assays for the target cytokine and GAPDH. Primers used to detect IFN-γ, TNF-α, COX-1, and COX-2 are described in Table 2. For these measurements, 5 µl of cDNA were added to reactions containing Power SYBR Green PCR Mix (Applied Biosystems) and forward and reverse primers (each at 200 nM final concentration) in a 25 µl reaction volume. When SYBR green was used to quantify cytokine gene expression, separate reactions were prepared with primers for mouse GAPDH (Table 2, used at 200 nM each). In all cases, RT-qPCR analysis consisted of 40 cycles of 15 s at 90°C and 60 s at 60°C. Quantification of target gene mRNA was normalized to GAPDH and expressed in arbitrary units as 2^-ΔCt^, where Ct is the threshold cycle and ΔCt = Ct(target) – Ct(GAPDH).

### Analysis of Inflammatory Cells in Bronchoalveolar Lavage Fluid

Mice were euthanized via pentobarbital overdose at the indicated time points. Lungs were lavaged three times with the same aliquot of 1 mL sterile PBS containing protease inhibitor (complete, Mini, EDTA-free tablets; Roche Applied Science). Cells in bronchoalveolar lavage fluid (BALF) were counted using a hemocytometer. When RNA was extracted from cells in BALF, the cells pelleted in a tabletop microcentrifuge at 17,000 x g for 10 min at 4°C and then resuspended in 0.5 mL of TRIzol® (Invitrogen). RNA was subsequently isolated according to the manufacturer’s protocol.

### Analysis of Cytokine Protein in Bronchoalveolar Lavage Fluid

The remaining cells in BALF were pelleted by centrifugation and supernatant was stored at -80°C. Cytokine protein concentrations in supernatant were determined by ELISA (Duoset Kits, R&D Systems) according to the manufacturer's protocol.

### Lung PGE_2_ Measurements

Lung tissue was suspended in CelLytic MT (Sigma-Aldrich) containing protease inhibitor (complete, Mini, EDTA-free tablets; Roche Applied Science) and 10 mM indomethacin (Sigma Aldrich) at a concentration of 100 mg lung tissue per 1 mL homogenization buffer. Tissue was homogenized (MagNA Lyser, Roche Applied Science) in 2 x 60 s cycles at high speed (6,000) with 90 s cooling between cycles. After homogenization, tissue was spun twice at 17,000 x g for 10 min at 4°C and supernatant was stored at -80°C until assayed. Samples were diluted in PGE_2_ enzyme immunoassay buffer and quantity of PGE_2_ was determined using PGE_2_ ELISA Kit (Enzo Life Sciences) according to the manufacturer’s protocol.

### Statistics

Analysis of data for statistical significance was conducted using Prism 3 for Macintosh (GraphPad Software, Incorporated). Differences between groups at multiple time points were analyzed using two-way analysis of variance (ANOVA) followed by Bonferroni's multiple comparison tests. Comparisons between two groups at a single time point were made using the Mann-Whitney rank sum test. *P* values less than 0.05 were considered statistically significant. 

## Results

### Induction of COX-2 expression and PGE_2_ production by MAV-1 in vivo

**Figure 1:**
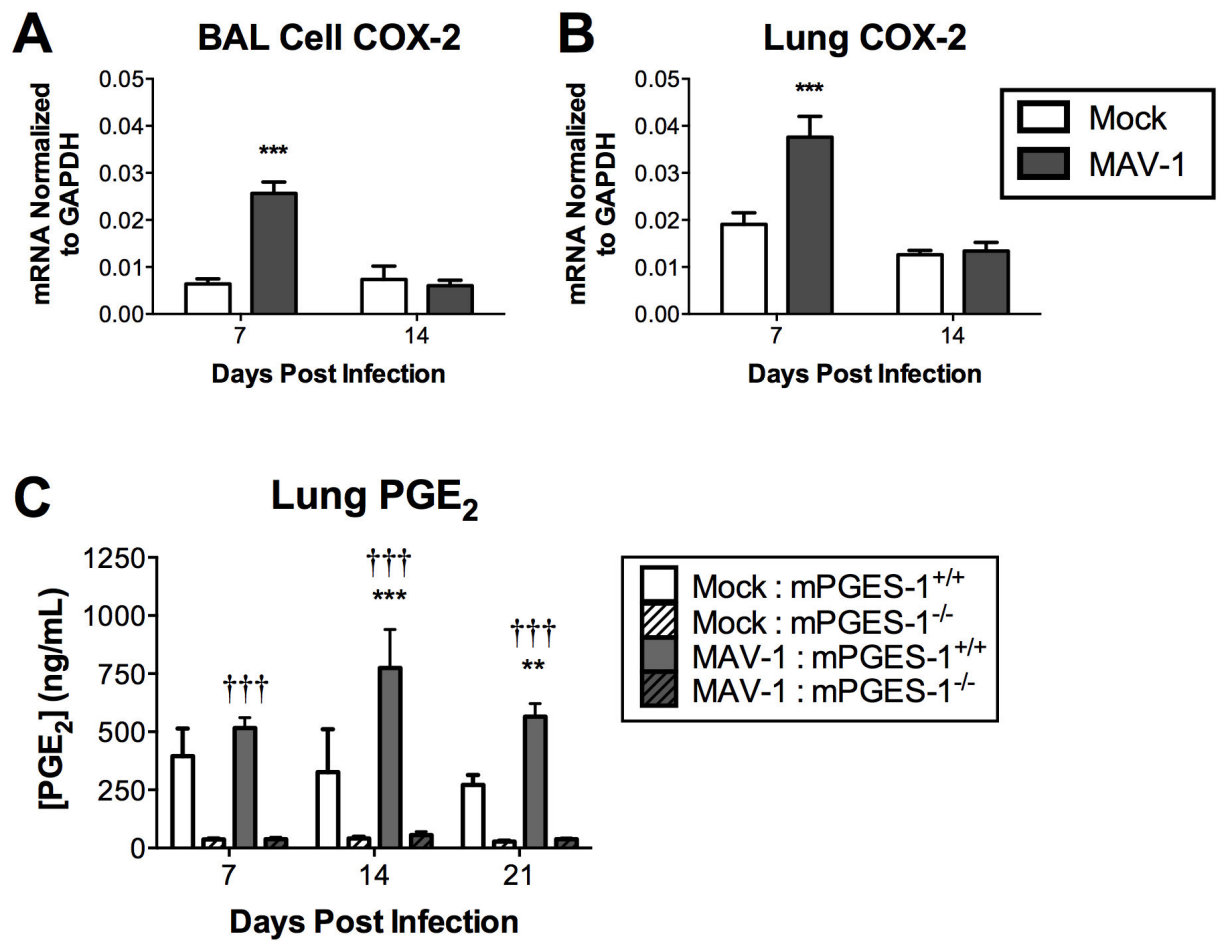
Induction of lung COX-2 expression and PGE_2_ production. Mice were infected i.n. with MAV-1 (grey bars) or mock infected (white bars) with conditioned media. A-B) RNA was extracted from BAL cells or lungs harvested at the indicated time points and RT-qPCR was used to quantify COX-2 expression, which is expressed in arbitrary units. C) ELISA was used to quantify PGE_2_ concentrations in lung homogenates from both mPGES-1^+/+^ and mPGES-1^-/-^ mice at the indicated time points. Combined data from n=8-9 (for BAL COX-2), n=5-23 (for lung COX-2) and n=3-5 (for ELISA) mice per group are presented as means ± S.E.M. Statistical comparisons were made using two-way ANOVA followed by Bonferroni’s multiple comparison tests. *P<0.05, **P<0.01 and ***P<0.001, comparing mock to MAV-1 for a given genotype. †††*P*< 0.001, comparing genotypes within the same condition.

To investigate whether MAV-1 respiratory infection induces COX-2 expression and PGE_2_ production *in vivo*, we infected wild-type (mPGES-1^+/+^) mice intranasally (i.n.) with MAV-1 and harvested bronchoalveolar lavage (BAL) cells and lung tissue at times corresponding to early infection (4 days post infection, dpi), the peak of viral replication at 7 dpi [34,35], and later times (14 and 21 dpi) corresponding to clearance of virus from the lungs. Because inflammatory stimuli, including infection with a variety of pathogens, are frequently associated with upregulated COX-2 expression [44-48], we first used reverse transcriptase quantitative real-time PCR (RT-qPCR) to measure COX-2 mRNA levels following MAV-1 infection. COX-2 mRNA was significantly increased in the lungs and BAL cells of infected mice compared to mock infected mice at 7 dpi and decreased to baseline levels seen in mock infected mice by 14 dpi (Figure 1A,B). Although it was detected in both mock infected and infected mice, COX-1 expression was not upregulated by MAV-1 infection (data not shown). PGE_2_ concentrations measured in lung homogenates steadily increased after infection, with significantly elevated levels at 14 and 21 dpi (Figure 1C, mPGES-1^+/+^ mice). These data demonstrate that acute MAV-1 infection increases COX-2 mRNA and induces PGE_2_ production in the lung. 

### Effects of EP2 deficiency on MAV-1 respiratory infection

**Figure 2:**
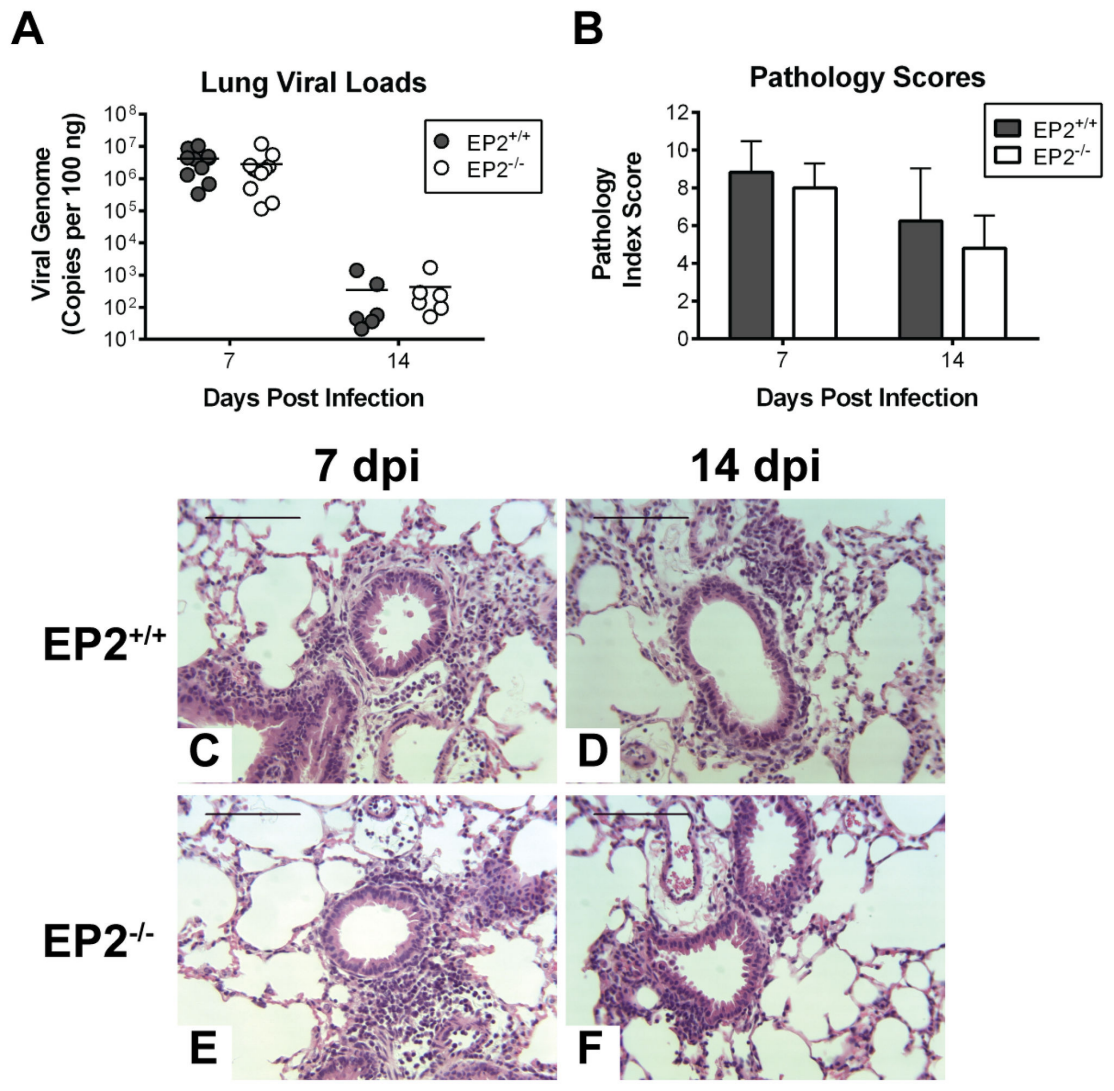
Effects of EP2 deficiency on MAV-1 respiratory infection. Mice were infected i.n. with MAV-1. A) DNA was extracted from lungs from EP2^+/+^ and EP2^-/-^ mice at the indicated time points. qPCR was used to quantify DNA viral loads, which are expressed as copies of MAV-1 genome per 100 ng of input DNA. Individual circles represent values for individual mice and horizontal bars represent means for each group. B) Pathology index scores were generated to quantify cellular inflammation. Combined data from 4 to 6 mice per group are presented as means ± S.E.M. C-F) Hematoxylin and eosin-stained sections were prepared from paraffin-embedded sections (bottom panels). Scale bars, 100 µm.

The physiological effects of PGE_2_ depend on its activation of four distinct cell membrane-associated G protein-coupled E prostanoid (EP) receptors [49]. PGE_2_ inhibits alveolar macrophage (AM) phagocytosis via EP2 activation and subsequent increases in cAMP [50], and PGE_2_ also inhibits bacterial killing by AMs and reactive oxygen intermediate generation by AMs in an EP2/EP4- and cAMP-dependent manner [51]. The inhibitory effects of PGE_2_ on host inflammatory responses have been linked to signaling through EP2 and EP4 [52], and PGE_2_ signaling through EP2 suppresses clearance from the lungs of *Pseudomonas aeruginosa* [47] and *Streptococcus pneumoniae* [49]. To determine whether PGE_2_ has a similar effect on control of MAV-1 infection or modulation of MAV-1-induced lung inflammation, we first studied acute MAV-1 respiratory infection in EP2-deficient (EP2^-/-^) mice. Following i.n. infection with MAV-1, no deaths occurred in either EP2^-/-^ or EP2^+/+^ controls. Lung viral loads were comparable in EP2^-/-^ and EP2^+/+^ mice at 7 dpi (Figure 2A), which we have previously described as the peak of viral replication in the lungs [34,35]. Viral loads were substantially less in both EP2^-/-^ and EP2^+/+^ mice at 14 dpi, with no significant differences between the groups at this time point.

 Acute MAV-1 respiratory infection induced a moderate pneumonitis in EP2^+/+^ mice, with the accumulation of inflammatory cells around airways and hypercellularity in alveolar walls by 7 dpi that decreased somewhat by 14 dpi (Figures 2C,D). We observed similar patterns of MAV‑1‑induced inflammation in the lungs of EP2^-/-^ mice at both 7 and 14 dpi (Figures 2E,F). Pathology index scores (Table 1) quantifying lung inflammation confirmed that there was not a significant difference between EP2^+/+^ and EP2^-/-^ mice at either time point (Figure 2B).

### Effects of mPGES-1 deficiency on MAV-1-induced lung inflammation

It is possible that redundancy of function between EP2 and EP4, which both mediate PGE_2_-induced increases in cAMP, accounted for the lack of differences seen between EP2^+/+^ and EP2^-/-^ mice. To capture the possible contributions of PGE_2_ to MAV-1 pathogenesis without regard to individual receptors, we used mice deficient in mPGES-1. This enzyme is responsible for the majority of the conversion of PGH_2_ to PGE_2_, so mPGES-1-deficient (mPGES-1^-/-^) mice are almost completely PGE_2_-deficient (Figure 1C and refs. 7,53). This strategy also allows us to assess whether PGE_2_ may influence MAV-1 infection via interactions with EP1 or EP3 receptors as well. Consistent with this, PGE_2_ levels in lung homogenates from mPGES-1^-/-^ mice were substantially lower than in mPGES‑1^+/+^ control mice and remained unchanged after MAV‑1 infection (Figure 1C). We did not detect any compensatory increase in mRNA levels of mPGES-2 or cPGES in mPGES-1^-/-^ mice compared to mPGES‑1^+/+^ controls at baseline before infection or at any time after infection (data not shown).

**Figure 3:**
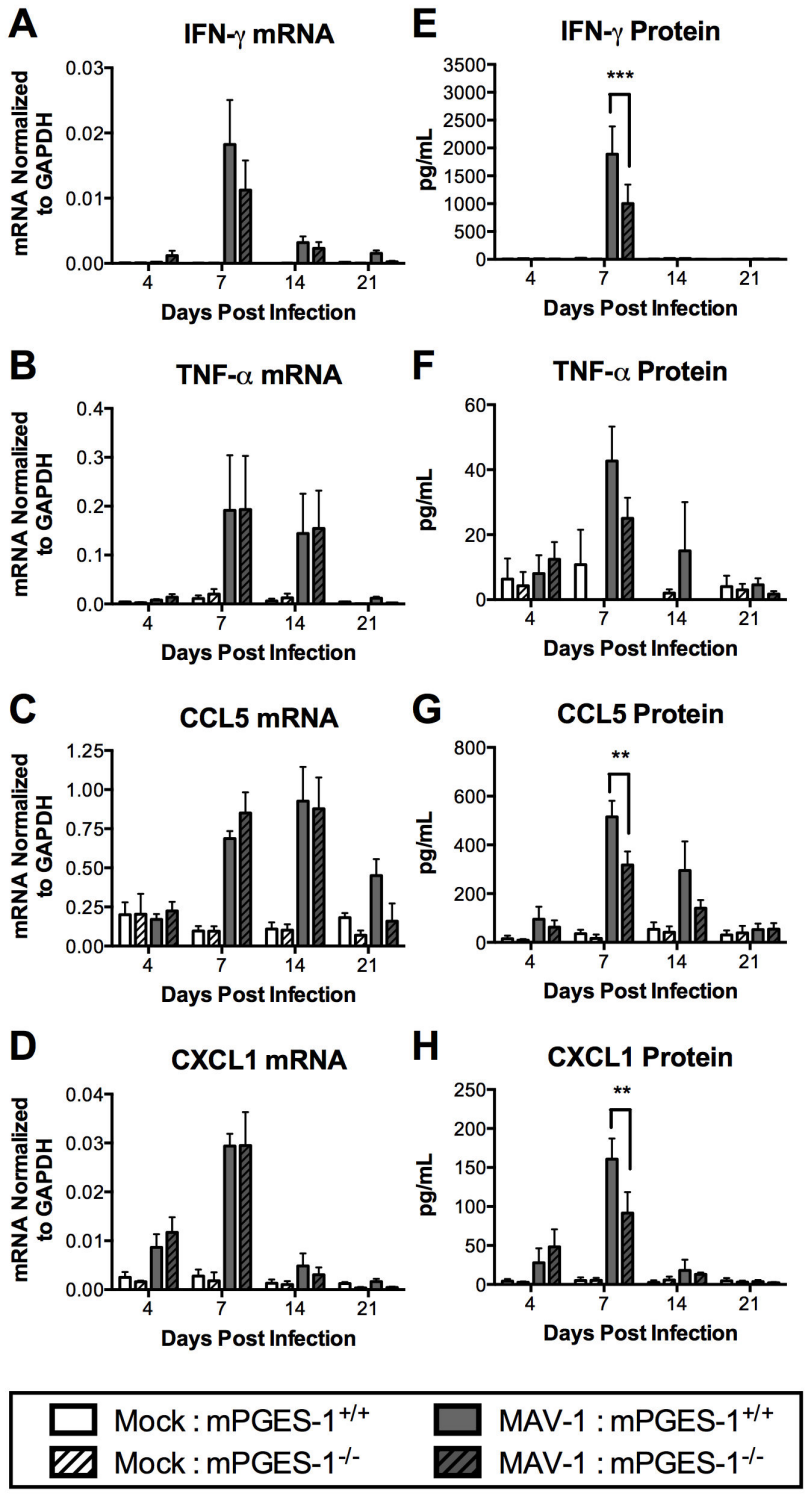
Effects of mPGES-1 deficiency on MAV-1-induced cytokine production. mPGES‑1^+/+^ and mPGES-1^-/-^ mice were infected i.n. with MAV-1 or mock infected with conditioned media. A-D) RNA was extracted from lungs harvested at the indicated time points and RT-qPCR was used to quantify cytokine expression, which is shown in arbitrary units. E-H) ELISA was used to quantify cytokine concentrations in BALF at the indicated time points. Combined data from 3 to 5 mice per group are presented as means ± S.E.M. Statistical comparisons were made using two-way ANOVA followed by Bonferroni’s multiple comparison tests. **P<0.01.

Decreased PGE_2_ production is associated with decreased virus-induced cytokine production following influenza virus infection of COX-2^-/-^ mice or mice treated with the COX-2 inhibitor celecoxib [30,54]. We hypothesized that PGE_2_ promotes virus-induced cytokine and chemokine production following MAV-1 infection. To determine whether PGE_2_ deficiency in mPGES-1^-/-^ mice affected MAV‑1-induced cytokine responses, we measured mRNA and protein levels of cytokines and chemokines that are commonly induced by MAV-1 infection [34,35]. At 7 dpi, IFN-γ mRNA was significantly increased in lungs of infected mice compared to mock-infected mice, although induction did not differ between mPGES‑1^+/+^ and mPGES-1^-/-^ mice (Figure 3A). MAV-1 infection induced similar increases of TNF-α mRNA in mPGES‑1^+/+^ and mPGES-1^-/-^ mice at 7 and 14 dpi (Figure 3B). At 7 and 14 dpi, lung CCL5 mRNA was significantly increased after infection, although the magnitude of induction was similar in mPGES‑1^+/+^ and mPGES-1^-/-^ mice (Figure 3C). The kinetics and magnitude of CXCL1 mRNA were similar in infected mPGES‑1^+/+^ and mPGES-1^-/-^ mice, with maximal induction occurring at 7 dpi (Figure 3D).

For each cytokine examined, peak induction of protein in BALF occurred at 7 dpi and protein levels then decreased over time, returning to baseline by 21 dpi. Peak IFN-γ protein concentrations were detected at 7 dpi in BALF from both infected mPGES‑1^+/+^ and mPGES-1^-/-^ mice, but the amount of IFN-γ protein was significantly less in mPGES-1^-/-^ mice than in mPGES‑1^+/+^ mice (Figure 3E). By 14 dpi, IFN-γ in both mPGES‑1^+/+^ and mPGES-1^-/-^ mice decreased to baseline levels. We did not detect changes of IL‑4 protein in BALF at any time point (data not shown), suggesting that PGE_2_ deficiency did not result in Th2 skewing following MAV-1 infection. Concentrations of TNF-α protein in BALF were also less in infected mPGES‑1^-/-^ mice than in mPGES‑1^+/+^ mice at 7 dpi, although this difference was not statistically significant (Figure 3F). TNF-α protein concentrations in BALF returned to baseline by 14 dpi. CCL5 protein induction was also lower in infected mPGES-1^-/-^ mice compared to infected mPGES‑1^+/+^ mice at 7 and 14 dpi, although the difference was only statistically significant at 7 dpi (Figure 3G). At 7 dpi, concentrations of CXCL1 protein in BALF were less in infected mPGES-1^-/-^ mice than in mPGES‑1^+/+^ mice (Figure 3H). By 14 dpi, CXCL1 decreased to baseline levels in both mPGES‑1^+/+^ and mPGES-1^-/-^ mice. 

**Figure 4:**
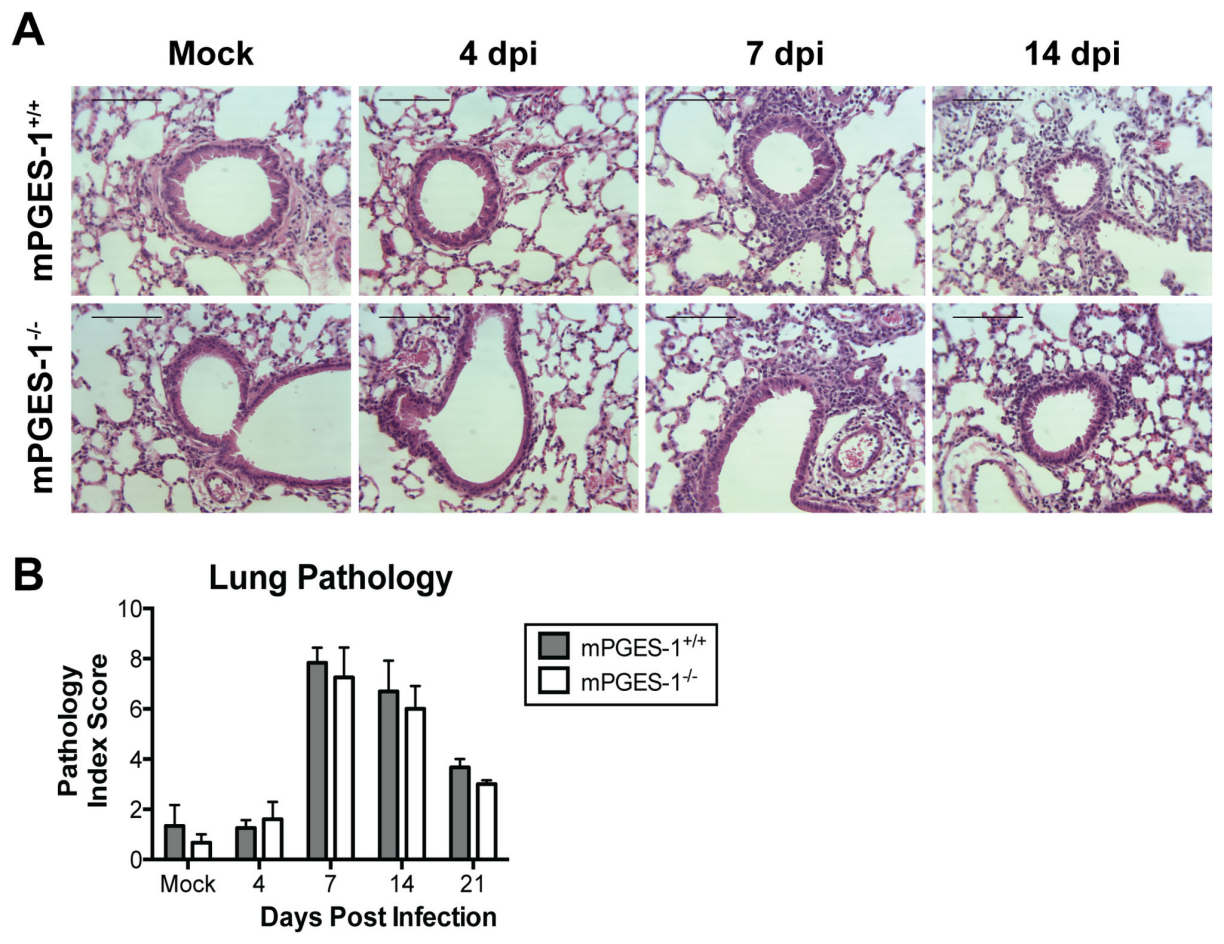
Effects of mPGES-1 deficiency on MAV-1-induced lung inflammation. mPGES-1^+/+^ and mPGES-1^-/-^ mice were infected i.n. with MAV-1 or mock infected with conditioned media. Lungs were harvested at the indicated time points. A) Hematoxylin and eosin-stained sections were prepared from paraffin-embedded sections. Scale bars, 100 µm. B) Pathology index scores were generated to quantify cellular inflammation. Combined data from 3 to 6 mice per group are presented as means ± S.E.M.

To determine whether these effects on proinflammatory cytokines and chemokines correlated with changes in other measures of virus-induced lung inflammation, we enumerated leukocytes in BALF obtained from mPGES‑1^+/+^ and mPGES-1^-/-^ mice after infection. There were no statistically significant differences between infected mPGES‑1^+/+^ and mPGES-1^-/-^ mice in the numbers or types of leukocytes in BALF at any time point examined (data not shown). Next, we evaluated MAV-1-induced cellular inflammation in the lungs of mPGES‑1^+/+^ and mPGES‑1^-/-^ mice. As we have previously described [34,35], we observed focal areas of inflammation surrounding medium and large airways, accompanied by scattered interstitial infiltrates in both mPGES‑1^+/+^ and mPGES-1^-/-^ mice (Figure 4A). Lung inflammation peaked at 7 dpi and became somewhat less pronounced by 14 dpi. By 21 dpi, cellular inflammation had largely resolved in both mPGES‑1^+/+^ and mPGES-1^-/-^ mice. We used pathology index scores (Table 1) to quantify lung inflammation. Pathology scores were greater in infected mice than in mock infected controls at 7 and 14 dpi, when inflammation was greatest (Figure 4B). There were no statistically significant differences in pathology index scores measured in mPGES‑1^+/+^ and mPGES-1^-/-^ mice at any time.

It is possible that mPGES-1 deficiency could result in shunting of the COX-derived intermediate PGH_2_ to other synthases such as the prostaglandin I_2_ (PGI_2_) synthase, leading to increased production of the next most abundant COX pathway product, PGI_2_. Because PGI_2_ signaling through the IP receptor also involves increases in intracellular cAMP, PGI_2_ overproduction could potentially compensate for PGE_2_ deficiency in our model. To determine whether this was the case, we measured concentrations of the PGI_2_ metabolite 6-keto-PGF1α in lung homogenates before and after infection. We observed small but insignificant increases of 6-keto-PGF1α in both mPGES‑1^+/+^ and mPGES-1^-/-^ mice after infection compared to mock infected mice. However, there were no significant differences between 6-keto-PGF1α concentrations in mPGES‑1^+/+^ and mPGES-1^-/-^ mice at any time point (data not shown). This suggests that PGI_2_ overproduction does not substantially compensate for any effect of PGE_2_ deficiency in mPGES‑1^-/-^ mice infected with MAV-1. 

### Effects of mPGES-1 deficiency on susceptibility to MAV-1

**Figure 5:**
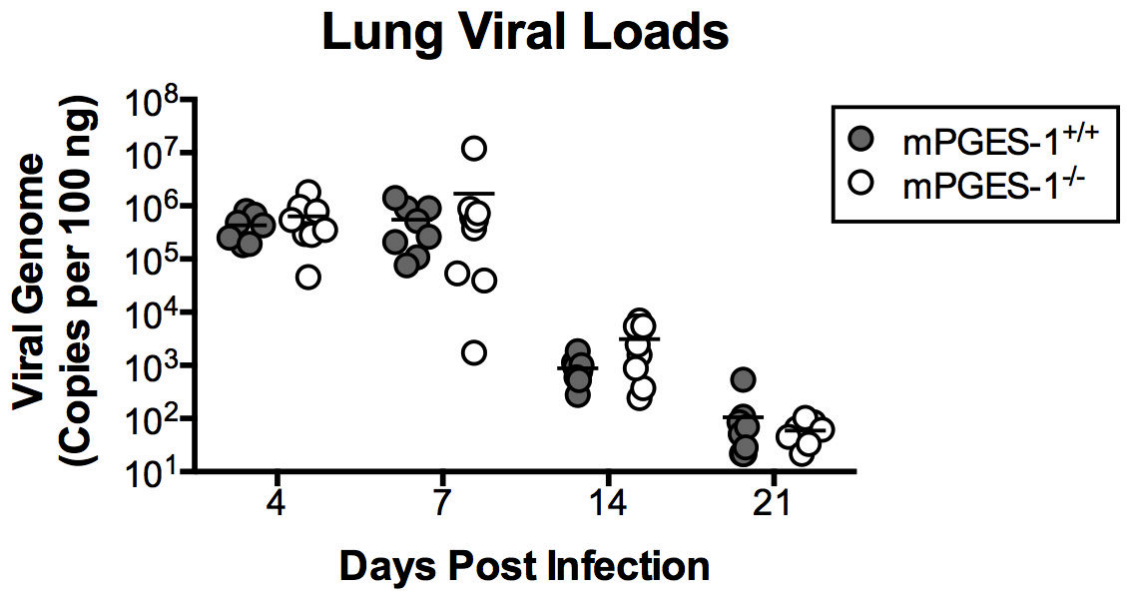
Effects of mPGES-1 deficiency on MAV-1 viral loads. mPGES-1^+/+^ and mPGES-1^-/-^ mice were infected i.n. with MAV-1 or mock infected with conditioned media. DNA was extracted from lungs harvested at the indicated time points. qPCR was used to quantify MAV-1 genome copies in lung DNA. DNA viral loads are expressed as copies of MAV-1 genome per 100 ng of input DNA. Individual circles represent values for individual mice and horizontal bars represent means for each group.

PGE_2_ deficiency in mPGES-1^-/-^ mice was associated with less production of IFN-γ and other cytokines in the airways of infected mice (Figure 3). To determine whether these differences correlated with increased susceptibility to MAV-1 infection, we used qPCR to quantify viral loads in the lungs and other target organs. Virus was detectable in the lungs by 4 dpi, and viral loads peaked at 7 dpi in both mPGES‑1^+/+^ and mPGES-1^-/-^ mice (Figure 5). Lung viral loads decreased substantially in both mPGES‑1^+/+^ and mPGES-1^-/-^ mice at 14 and 21 dpi, consistent with clearance of virus from the lungs in both groups (Figure 5). There were no statistically significant differences in lung viral loads measured in mPGES‑1^+/+^ mice compared to mPGES‑1^-/-^ mice at any time point. Likewise, there were no statistically significant differences in viral loads measured in the brains and spleens of mPGES‑1^+/+^ mice compared to mPGES-1^-/-^ mice at any time point (data not shown). Collectively, these data suggest that PGE_2_ deficiency does not affect the control of viral replication in the lungs during acute infection, clearance of virus from the lungs, or dissemination of virus to other target organs.

### Effect of the nonselective COX inhibitor indomethacin on MAV-1 infection

**Figure 6:**
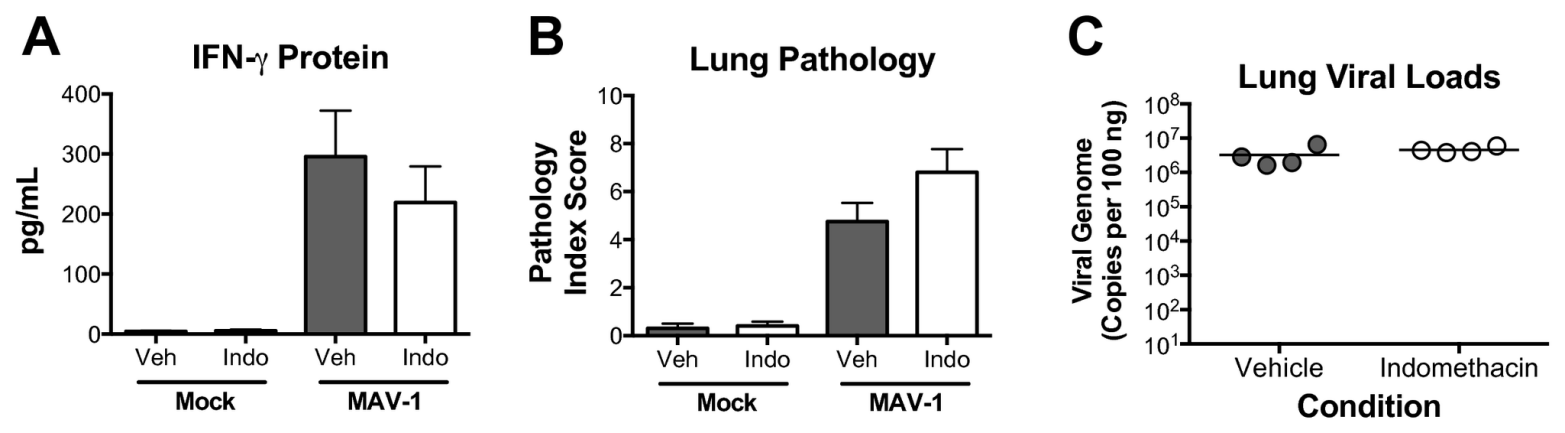
Effects of COX inhibition on MAV-1 respiratory infection. Wild type mice were infected i.n. with MAV-1 or mock infected with conditioned media. Mice were treated daily with indomethacin (1.2 mg/kg given i.p.) or vehicle control until samples were harvested at 7 d.p.i. A) ELISA was used to quantify IFN-γ concentrations in BALF. Combined data from 4 to 5 mice per group are presented as means ± S.E.M. B) Pathology index scores were generated to quantify cellular inflammation in lungs. Combined data from 4 to 5 mice per group are presented as means ± S.E.M. C) DNA was extracted from lungs and qPCR was used to quantify MAV-1 genome copies in lung DNA. DNA viral loads are expressed as copies of MAV-1 genome per 100 ng of input DNA. Individual circles represent values for individual mice and horizontal bars represent means for each group.

To determine whether COX-derived products other than PGE_2_ contribute to MAV-1-induced inflammatory responses, we infected mice i.n. with 10^5^ pfu MAV-1 and treated mice daily with an i.p. injection of indomethacin as previously described [55] and then harvested samples at 7 d.p.i. Indomethacin treatment reduced lung PGE_2_ concentrations by approximately 30% in infected mice (data not shown). Unlike our findings in mPGES-1^-/-^ mice, treatment of MAV-1-infected mice with indomethacin did not affect virus-induced production of IFN-γ, CXCL1, CCL5, or TNF-α (Figure 6A and data not shown). Likewise, indomethacin did not affect the development of lung pathology after MAV-1 infection (Figure 6B) or MAV-1 lung viral loads (Figure 6C).

### Adaptive immunity to MAV-1 is not substantially affected by PGE_2_ deficiency

**Figure 7:**
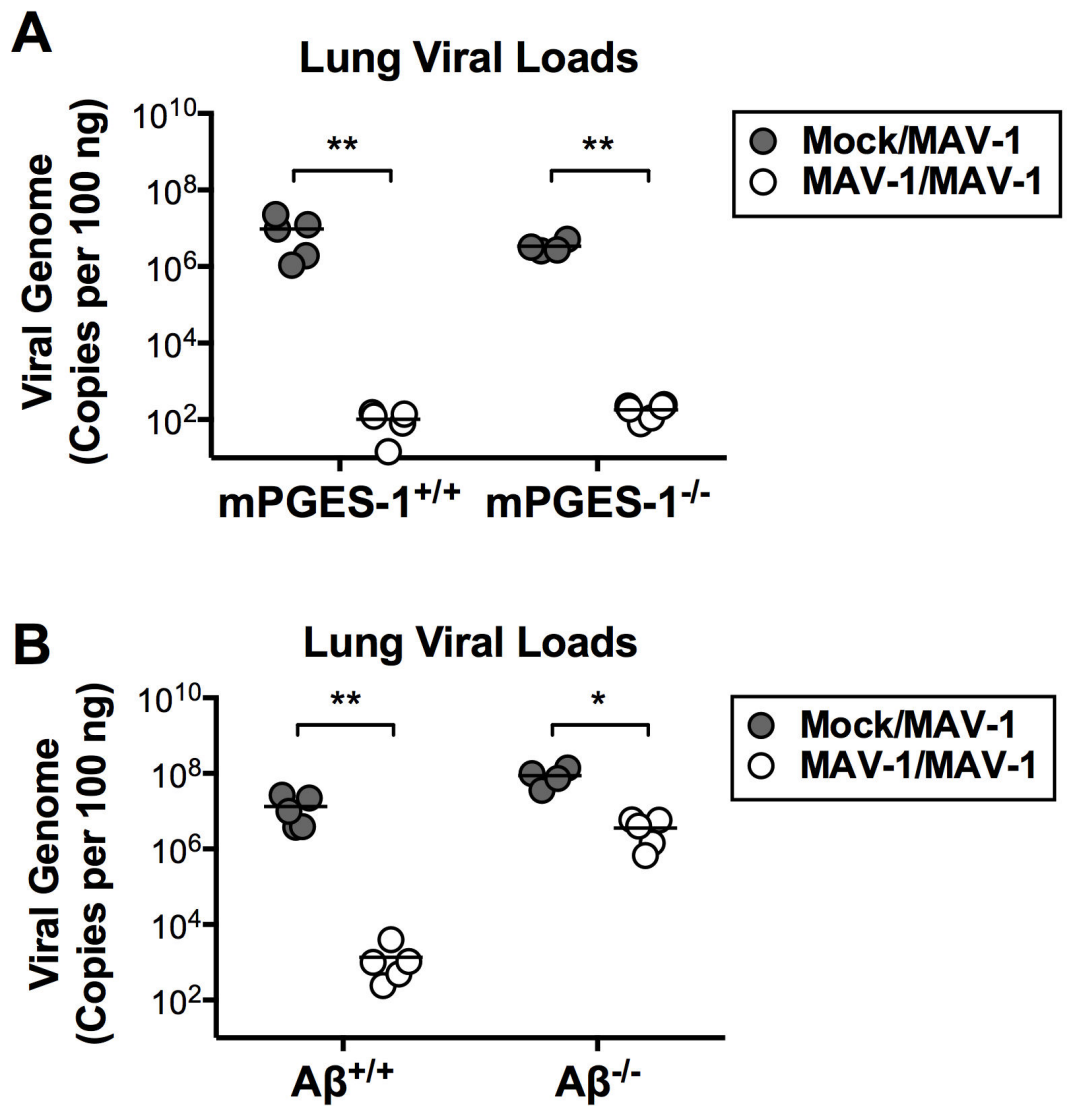
Protective immunity to MAV-1 infection. A) mPGES-1^-/-^ and B) Aβ^-/-^ mice, along with appropriate mPGES-1^+/+^ and Aβ^+/+^ controls, were infected i.n. with MAV-1 or mock infected with conditioned media. At 28 dpi, mice were re-infected i.n. with MAV-1 and lungs were harvested at 7 dpi. DNA was extracted from lungs and qPCR was used to quantify DNA viral loads, which are expressed as copies of MAV‑1 genome per 100 ng of input DNA. Individual circles represent values for individual mice and horizontal bars represent means for each group. Statistical comparisons were made using the Mann-Whitney rank sum test for differences between conditions within a given genotype. *P<0.05 and **P<0.01.

PGE_2_ has a variety of effects on T and B cell function that are likely to affect the development of adaptive immunity and subsequent protection from secondary infection. Because of the various effects of PGE_2_ on T and B lymphocyte function, we reasoned that PGE_2_ deficiency might inhibit appropriate adaptive immune responses to MAV-1 infection. To examine this, we infected or mock infected mPGES‑1^+/+^ and mPGES-1^-/-^ mice i.n. with 10^5^ p.f.u. of MAV-1 and then rechallenged them with virus or conditioned media at 28 dpi. We measured lung viral loads at 7 days after the second challenge, using protection (lower lung viral loads following rechallenge) as a marker of adaptive immune function. Virus was readily detectable in mPGES‑1^+/+^ mice that were originally mock infected and then infected with virus 28 days later (Figure 7A). mPGES‑1^+/+^ mice that were initially infected with virus and then rechallenged with virus at 28 dpi had viral loads that were significantly lower than viral loads measured in mice that were initially mock infected and then infected with virus 28 days later (Figure 7A). This suggests that mPGES‑1^+/+^ mice were capable of generating a protective adaptive immune response. When we rechallenged mPGES‑1^-/-^ mice, we observed protection equivalent to that observed in mPGES‑1^+/+^ mice (Figure 7A).

To verify that this experimental design could demonstrate a difference in adaptive immune responses, we performed a similar rechallenge experiment using Aβ^-/-^ (MHC II-deficient) mice. Following primary infection, lung viral loads in Aβ^-/-^ mice were approximately 1 log unit higher than in Aβ^+/+^ mice at 7 dpi (Figure 7B). While lung viral loads were slightly lower in Aβ^-/-^ mice rechallenged with virus than in Aβ^-/-^ mice following primary infection, this difference was substantially less than the corresponding difference in Aβ^+/+^ mice (Figure 7B). As expected, the data from these rechallenge experiments indicate that MHC II (and thus CD4 T cells) are required for the development of protective immunity to MAV‑1. Although subtle effects of PGE_2_ deficiency on specific aspects of T or B lymphocyte function remain possible, these data suggest that PGE_2_ does not make substantial contributions to adaptive immune responses that are required for protection against MAV-1 respiratory infection.

## Discussion

The expression of COX-2 and production of PGE_2_ increases in response to acute respiratory infection with several viruses [21-29]. Previous studies have typically used COX inhibition or COX-deficient animals to study contributions of PGE_2_ to viral pathogenesis. Because these strategies affect all COX-derived mediators, specific roles played by PGE_2_ during viral respiratory infection remain unclear. In this study, we demonstrate that acute MAV-1 respiratory infection also induces COX-2 expression and PGE_2_ production in the lungs. PGE_2_ regulates immune function in many ways that could potentially affect viral pathogenesis. Inhibition of PGE_2_ production during respiratory viral infection with RSV or influenza leads to decreased pro-inflammatory cytokine production and decreased pulmonary inflammation [22,30]. Based on these previous studies, we hypothesized that PGE_2_ coordinates inflammatory responses during adenovirus respiratory infection. Using mPGES-1-deficient mice, we demonstrated that PGE_2_ promoted the production of some inflammatory cytokines during MAV‑1 infection. However, we were surprised to find no evidence that PGE_2_ regulated viral replication, inflammatory cell accumulation, inflammatory cell composition, or development of protective adaptive immune responses.

Our results differ from what has been observed with other respiratory viruses. For example, inhibition of PGE_2_ production during influenza or RSV infection has significant effects on virus-induced inflammatory responses. During influenza infection, treatment of mice with the COX-2 inhibitor celecoxib suppresses virus-induced production of proinflammatory cytokines in the lungs, although it does not affect viral titers or disease severity [54]. Treatment of influenza-infected mice with COX inhibitors results in improved lung function and reduced immunopathology [56]. In our study, treatment with the nonselective COX inhibitor indomethacin did not affect virus-induced lung pathology or cytokine production (Figure 6A-B), despite decreasing PGE_2_ levels. This lack of effect again suggest differences between the role of PGE_2_ and other COX-derived mediators in MAV-1 pathogenesis compared to other viruses, although it may also reflect an incomplete suppression of PGE_2_ production by drug treatment. Genetic COX‑2 deficiency has a more pronounced effect on influenza-induced disease than does pharmacologic inhibition, because COX-2^-/-^ mice have reduced mortality, inflammation and cytokine responses after influenza infection compared to wild-type control mice [30]. Treatment of RSV-infected airway epithelial cells with the COX-2 inhibitors NS-398 or celecoxib reduces production of virus particles and proinflammatory cytokines *in vitro* [21], although COX inhibition does not appear to significantly affect RSV replication *in vivo*. Similar to the effects of COX inhibition in influenza-infected mice, COX inhibition results in decreased lung pathology in RSV-infected cotton rats [22]. 

Our results showing reduced production of proinflammatory cytokines after MAV-1 infection of mPGES-1-deficient mice are similar to the effects of COX inhibition during influenza or RSV infection. This supports a role for PGE_2_ in promoting proinflammatory cytokine production in the lung during viral infection. However, unlike studies using COX inhibition during influenza or RSV infection, we did not observe a substantial effect of PGE_2_ deficiency on MAV‑1-induced lung pathology. This suggests that the effects on lung pathology observed with COX inhibition during influenza or RSV infection could be due to decreased production of another COX-derived eicosanoid such as thromboxane, PGD_2_, PGF_2_, and PGI_2_. Interestingly, levels of PGE_2_ in influenza-infected COX-2^-/-^ mice are equivalent to levels measured in infected COX-2^+/+^ control mice [30], further supporting the possibility that the effects of COX-2 deficiency on influenza pathogenesis may not be solely due to decreased production of PGE_2_. We typically observe host responses to acute MAV-1 respiratory infection that are generally similar to those seen with RSV and influenza infection. However, RNA viruses such as RSV and influenza are likely to interact with different pattern recognition receptors than a DNA virus such as MAV-1 or the human adenoviruses. It is possible that subtle differences in the mechanisms underlying the induction of inflammatory responses by these viruses, along with differential effects of PGE_2_ or other eicosanoids on those responses, could account for differences between our results with MAV-1 and those with RSV and influenza.

We used mice deficient in mPGES-1 to specifically characterize the effects of reduced PGE_2_ production on viral replication and host responses to primary MAV-1 infection. Although overproduction of other prostaglandins in the absence of mPGES-1 occurs in some models using mPGES-1-deficient mice [7,53,57,58], we did not detect significant overproduction of PGI_2_ metabolites in mPGES-1^-/-^ mice at baseline or following infection with MAV-1. Therefore, we do not believe that overproduction of other prostaglandins compensated for PGE_2_ deficiency in our experiments, reducing any potential effect on MAV-1 pathogenesis. It is possible that other mediators not measured, such as leukotrienes, could be compensating for PGE_2_ deficiency in our model. The use of mPGES‑1^-/-^ mice instead of pharmacologic inhibition of PGE_2_ production allowed us to study the effects of almost complete PGE_2_ deficiency. COX or mPGES-1 inhibitors do not achieve complete inhibition of enzymatic activity by COX or mPGES‑1, whereas mPGES-1^-/-^ mice have a complete loss of mPGES-1 activity and are therefore more completely PGE_2_-deficient [7,53]. It is possible that mice genetically deficient in mPGES-1 could have functional differences in the development of the immune system that we have not defined in our experiments, possibly establishing compensatory pathways to overcome any defects that would have been the result of PGE_2_ deficiency. 

We have previously demonstrated that MAV-1 induces a Th1 response in the lungs of infected mice, with significant upregulation of IFN-γ that peaks at 7 dpi [35]. Some reports have suggested that PGE_2_ promotes a Th2-polarized environment by suppressing production of the Th1 cytokines IFN-γ and IL-12 *in vitro* [12,13]. However, nanomolar concentrations of PGE_2_ enhance Th1 cytokine secretion and differentiation *in vivo* [14,15]. We demonstrated less induction of IFN-γ in mPGES‑1^-/-^ mice infected with MAV-1, supporting the notion that PGE_2_ could contribute to an appropriate Th1 response to MAV-1 infection. The decreased IFN-γ response observed in mPGES-1^-/-^ mice is likely due to a decrease in IFN-γ production by CD4^+^ and CD8^+^ T cells, as these cell types are the major producers of IFN-γ during MAV-1 respiratory infection (Mary McCarthy and Jason Weinberg, unpublished data). Viral loads in mPGES-1^-/-^ mice did not differ from those of mPGES‑1^+/+^ mice at 7 dpi despite less induction of IFN-γ in mPGES-1^-/-^ mice. We have previously demonstrated that IFN-γ does play some role in the control of viral replication [35], but results from that study and from the present experiments suggest that other factors are likely able to compensate for IFN-γ deficiency to control MAV-1 replication in the lung 

In addition to its contributions to T cell polarization, PGE_2_ plays an important role in promoting antibody synthesis and isotype switching [16-20]. Due to the potential effects of PGE_2_ on T cell polarization and B cell antibody responses, we hypothesized that PGE_2_ deficiency might inhibit appropriate adaptive immune responses to MAV-1 infection. Total serum IgG levels were similar in mPGES-1^+/+^ and mPGES-1^-/-^ mice (data not shown), suggesting that total antibody production in response to MAV-1 infection is unaffected by the absence of PGE_2_. It is possible that virus-specific antibody production and virus-specific T cell functions were altered by PGE_2_ deficiency. However, the results of our rechallenge experiments, in which both mPGES-1^+/+^ and mPGES‑1^-/-^ mice were protected by prior infection, suggest that PGE_2_ is not likely to substantially affect the development of protective immune responses to MAV-1. 

 COX inhibitors such as acetaminophen and ibuprofen are frequently used to alleviate fever and other symptoms in patients with respiratory infections. Decreases in RSV- and influenza-induced inflammation in animals treated with COX inhibitors or genetically deficient in COX-2 suggest that modulation of virus-induced PGE_2_ production may have other benefits for patients with infections caused by some viruses. Our results with MAV-1 infection of PGE_2_-deficient mice suggest that PGE_2_ promotes MAV-1-induced cytokine production but does not have a dramatic effect on MAV-1-induced lung inflammation or control of viral replication. A more generalized inhibition of eicosanoids in addition to PGE_2_ may be necessary to achieve more pronounced effects on virus-induced inflammatory responses. Ultimately, this may provide an attractive approach to limiting damage caused by virus-induced inflammation without having a substantial effect on the control of viral infection by host immune responses.
